# ITC-derived binding affinity may be biased due to titrant (nano)-aggregation. Binding of halogenated benzotriazoles to the catalytic domain of human protein kinase CK2

**DOI:** 10.1371/journal.pone.0173260

**Published:** 2017-03-08

**Authors:** Maria Winiewska, Ewa Bugajska, Jarosław Poznański

**Affiliations:** Department of Biophysics, Institute of Biochemistry and Biophysics, Polish Academy of Sciences, Warszawa, Poland; Russian Academy of Medical Sciences, RUSSIAN FEDERATION

## Abstract

The binding of four bromobenzotriazoles to the catalytic subunit of human protein kinase CK2 was assessed by two complementary methods: Microscale Thermophoresis (MST) and Isothermal Titration Calorimetry (ITC). New algorithm proposed for the global analysis of MST pseudo-titration data enabled reliable determination of binding affinities for two distinct sites, a relatively strong one with the K_d_ of the order of 100 nM and a substantially weaker one (K_d_ > 1 μM). The affinities for the strong binding site determined for the same protein-ligand systems using ITC were in most cases approximately 10-fold underestimated. The discrepancy was assigned directly to the kinetics of ligand nano-aggregates decay occurring upon injection of the concentrated ligand solution to the protein sample. The binding affinities determined in the reverse ITC experiment, in which ligands were titrated with a concentrated protein solution, agreed with the MST-derived data. Our analysis suggests that some ITC-derived K_d_ values, routinely reported together with PDB structures of protein-ligand complexes, may be biased due to the uncontrolled ligand (nano)-aggregation, which may occur even substantially below the solubility limit.

## Introduction

Protein kinase CK2 (formerly known as casein kinase 2) is the subject of a common interest due to its key role in signaling pathways controlling numerous cellular functions. It is a pleiotropic kinase with over 200 substrates identified to date [1]. Protein kinase CK2 has become a therapeutic target in cancer treatment. Although, CK2 does not appear to be oncogenic itself, there is a statistically significant correlation between malignancy and its abnormally high activity in cancer cells [2].

**Fig 1:**
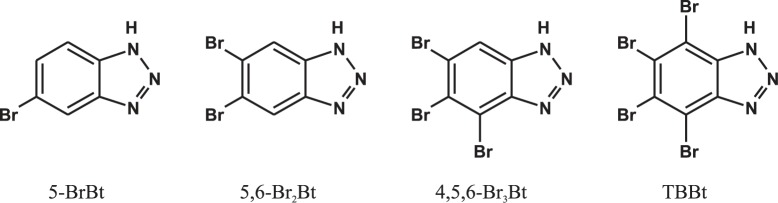
Schematic structures of the studied bromobenzotriazoles.

Many potent inhibitors of CK2 have been reported so far, dozens of them were derived from either tetrabromobenzotriazole (TBBt) or tetrabromobenzimidazole (TBBz) [3–5]. Most of the compounds act as ATP-competitive inhibitors targeting the ATP binding site of the catalytic subunit, CK2α [6–8]. The set of thermodynamic data for CK2α-ligand complexes is constantly increasing. We have studied thermodynamic properties of the CK2α interaction with a series of four benzotriazole derivatives variously brominated on the benzene ring. Some of these compounds (Fig 1) displayed inhibitory activity comparable to that of the reference TBBt [9].

Although, the studied ligands are not potent CK2 inhibitors, they are a promising starting point for further modifications. Moreover, this series of structurally close compounds with different physico-chemical properties (size, aqueous solubility, pK_a_ for dissociation of the triazole proton), serves as a useful probe for sampling contribution of various types of intermolecular interactions, including recently defined halogen bonding, to the free energy of ligand binding [10]. According to our knowledge, there are numerous data for individual ligands, but very limited thermodynamic studies for series of closely related low-mass ligands [11–13]. Decomposition of thermodynamic parameters determined experimentally for series of ligands should provide very useful information for further drug design approaches. Interestingly, although the effect of possible ligand aggregations is rarely studied, it may substantially affect apparent inhibitory activity [14–20]. Huang and Lau have recently analyzed the sources of the possible biases in ITC studies on protein interactions with various types of nano-particles, demonstrating that protein samples should be preferably used as a titrant [21].

Isothermal Titration Calorimetry (ITC) and Microscale Thermophoresis (MST) are the experimental techniques commonly used for determination of binding affinities for biomolecules. While ITC is a well known technique routinely used to study the thermodynamics of CK2-inhibitor interactions [5, 22], MST is a relatively new method, but there are already some studies concerning CK2 [13, 23].

ITC provides a complete set of thermodynamic parameters describing ligand binding. It is the most accurate direct method for determination of the enthalpy of a reaction under isothermal and isobaric conditions. However it must be noted that due to the heat transfer inside the calorimeter, the observed signal (i.e. calorimetric raw response) is not proportional to the heat power released upon mixing reagents. Instead, it represents a Laplace transform that convolutes the signal with the response profile of the calorimeter [24] (13 s response time for NanoITC). Moreover, some requirements must be satisfied to obtain reliable results, which limits the application of the method [21, 25]. The required concentrations of reactants should match the range optimal for the binding affinity of the studied species [26, 27]. Moreover, the ligand concentration combined with the injection volume must result in a measurable heat effect, what even for enthalpy-driven reactions may be hard to meet for the reagents with limited availability or solubility [28–30]. The latter limitation may however be overcome by application of the reverse ITC experiment, in which the macromolecule is used as a titrant instead of the small-mass ligand [31]. It should also be noted that there are very few studies in which both modes of calorimetric titrations were applied in parallel [32, 33] or accompanied by alternative experimental techniques [34, 35].

Microscale Thermophoresis (MST) is an alternative approach that allows the direct estimation of binding affinities, which do not fall within the limitations described above. The strong advantage of the MST lies in low sample consumption, since the reactants are placed in micro-capillaries. Moreover, the applicable concentration range is substantially wider than for ITC, since the fluorescence signal can be monitored with higher sensitivity than heat effect. Commonly, the concentration of the non-titrated fluorescent binding partner is close to the expected value of the dissociation constant, K_d_, while the maximal concentration on the non-fluorescent partner exceeds the K_d_ value 10–20 fold [36]. The major disadvantage of MST is the use of fluorescent marker that may affect the studied interactions, which can however be overcome with a hardware enabling label-free approach. Aside from the above limitations, the MST differ from ITC in the sample preparation protocol, which in MST implies preincubation of the reactants, while in ITC the heat release is measured immediately after mixing of the reagents.

Herein we present the new method of global analysis of MST data used to assay the binding of four halogenated benzotriazoles by the catalytic subunit of human protein kinase CK2. The analysis enabled reliable study of two binding events. The stronger binding site has also been characterized by ITC, and the possible sources of discrepancies between MST- and ITC-derived dissociation constants were carefully analyzed. The obtained results strongly suggest the necessity of verification of ITC data by alternative methods. We demonstrate that, despite the limitations of the two methods, MST and ITC are complementary and together allow for determination of reliable values of thermodynamic parameters.

## Material and methods

### Expression and purification of hCK2α

The catalytic subunit of human CK2, hCK2α, was expressed and purified according to the method described previously [13].

### Synthetic procedures

Reagents and solvents (analytical grade), purchased from Sigma Aldrich, Chempur, Avantor and Merck, were used without further purification. Brominated ligands were prepared according to previously reported procedures [13, 37, 38], and their identity was confirmed by ^13^C NMR and mass spectrometry.

### Sample preparation

The protein samples of the required hCK2α concentration were prepared in 25 mM Tris–HCl (pH 8, 0.5 M NaCl) and 2% (v/v) DMSO concentration, which was previously used in enzymatic assays [9, 37, 38]. For MST experiments the protein sample additionally contained 5 mM β-mercaptoethanol to prevent sticking of protein to capillaries.

Ligands for all experiments, (ITC, MST, UV-Vis and DLS) were initially dissolved in DMSO, and the appropriate amounts of stock solutions were then diluted with 25 mM Tris–HCl (pH 8, 0.5 M NaCl) to obtain the required ligand concentration with a final 2% DMSO content. It should be stressed that the solvent composition of protein and ligand samples were always identical, so that the protein and ligand concentrations were the only variable factors.

### Microscale Thermophoresis (MST)

The hCK2α sample was initially labeled with the commercially available NT-647 dye, using NanoTemper Protein Labeling Kit RED. The concentration of the labeled protein was kept constant at ~100 nM, while the ligand to protein concentration ratio was in the range of 0.01 to 10^5^ nM. The samples were loaded into K002 Monolith™ NT.115 Standard Treated Capillaries, which after 10 min incubation time were analyzed using the Monolith NT.115 (NanoTemper Technologies). All obtained data, contrary to those previously reported for the same ligands [13], were analyzed according to the model of two independent binding sites. The algorithm for one-type of binding sites implemented in standard NanoTemper Software was found insufficient, since in numerous MST curves two well-separated inflection points were identifiable [13]. The numerical model was based on the cubic equation describing the concentration-dependent equilibrium of the *apo* form of the protein, free ligand, and their 1:1 and 1:2 complexes [39]

$$K_{1}\cdot K_{2}\cdot {\left[L\right]}^{3}+(K_{1}+K_{1}\cdot K_{2}\cdot (2\cdot P_{0}\;\text{-}\;L_{0}))\cdot {\left[L\right]}^{2}+(1+K_{1}\cdot (P_{0}\;\text{-}\;L_{0}))\cdot [L]=L_{0}$$ (1)

where P_0_ and L_0_ are the total concentrations of the protein and ligand, respectively; K_1_ and K_2_ are the dissociation constants for 1:1 and 1:2 complexes; and [L] is the concentration of the free ligand. The above equation can be resolved by roots against [L] (Cardano’s derivation of the cubic formula, first time published: G. Cardano *Ars magna* 1545), and the concentration of the *apo* protein, [P], was further calculated as

$$[P]=P_{0}/\left(1+K_{1}\cdot [L]+K_{1}\cdot K_{2}\cdot {\left[L\right]}^{2}\right)$$ (2)

Concentrations of 1:1 and 1:2 complexes equal K_1_∙[P]∙[L] and K_1_∙K_2_∙[P]∙[L]^2^, respectively. The model was implemented in Origin 9.0 package (www.originlab.com). Dissociation constants, K_1_ and K_2_, were estimated globally for a series of 6 MST pseudo-titration experiments, while contributions of three protein forms (i.e. *apo*, and 1:1 and 1:2 complexes) to the observed MST signal were fitted individually for each experiment (S1 Fig).

### Isothermal Titration Calorimetry (ITC)

All ITC measurements were carried out at 25°C with the Nano ITC calorimeter (TA Instruments), using 250 rpm stirring and 1000 s delay between succeeding injections to the sample cell (950 μl). The first “technical” injection of a reduced volume (4–5 μl) was followed by 12 injections of 20 μl, however 16x15 μl or 10x25 μl schemes were occasionally used. The concentration of reagents varied depending on their affinities and experiment type. Thus 20–200 μM ligand solution was used as the titrant, but only 4–10 μM when placed in the sample cell. Accordingly, the sample cell was filled with 4–30 μM hCK2α solution, while 20–40 μM protein solution was placed in the syringe. For each ligand at least two titration experiments were done with different reagent concentrations, and occasionally with optimized injection volume. The data shown in the manuscript were collected during two one-week sessions using the same protein stock, but results of additional control experiments remained consistent with the presented data. The resulting data were processed with the standard NanoAnalyze software package (version 3.5.0), assuming the model of one type of binding sites of the apparent stoichiometry close to 1:1. The automatically adjusted integration regions were used to minimize the impact of researcher’s arbitrary decision. The uncertainty of all determined values was estimated as the half-width of 67% confidence interval.

The model of two types of binding sites was also fitted to the ITC data, but the optimization procedure has not converged, since the solubility limits disabled the saturation of the weekly binding site. This problem was partially overcome by the combined approach, in which both K_d_ values were constrained to those determined with MST.

### Dynamic light scattering (DLS)

All DLS experiments were carried out at 25°C with the DynaPro NanoStar 192-DPN apparatus (Wyatt Technology) equipped with 661 nm laser. The autocorrelation function for the light scattered by 5-BrBt or TBBt buffered solution placed in Eppendorf disposable cuvettes (50–2000 μl) was measured at various solute concentrations in the range of 0.5 μs– 0.1 s, and further decomposed to the distribution of particle size (ranging from 0.18 nm to 2.5 μm) using Dynamics software (Wyatt Technology, ver. 7.0.2.7). Solutions with 2% (v/v) DMSO content were prepared in 25 mM Tris-HCl (pH 8, 0.5 M NaCl) filtered with 0.22 μm pore size syringe filter. All samples were centrifuged (9000 g) for 3 minutes immediately before the experiment. For each sample a series of at least 30 repetitions, 10 s each, has been collected, and those with abnormally high SOS function and/or with highly fluctuating SLS signal were removed from the analysis. All remaining data have been averaged to obtain the autocorrelation function (ACF) of a reasonable quality. This procedure was found indispensable for diluted TBBt solutions, in which large aggregates have rarely scattered light crossing the laser beam. Consequently, extreme fluctuations of ACF precluded direct analysis of the time-evolution of TBBt aggregates. However, the raw SLS signal was found applicable to estimate the rate of aggregate disintegration.

### UV-VIS spectroscopy

The absorption spectra (200–500 nm) were collected at 25°C using Varian Cary 50 UV-VIS spectrophotometer equipped with 1 cm quartz cells. The initial buffer solution was manually titrated with the 851 μM TBBt solution to record spectra for increasing solute concentration. For each sample UV-VIS spectrum was collected four times, every 20 s including 15 s acquisition time, starting 5 s after solute dilution. The changes in the absorption spectrum of 40 μM TBBt sample, prepared just before the experiment, were additionally tracked for an hour to follow possible disintegration of soluble aggregates.

## Results and discussion

### Thermodynamic parameters of the CK2α-inhibitor interaction

**Fig 2:**
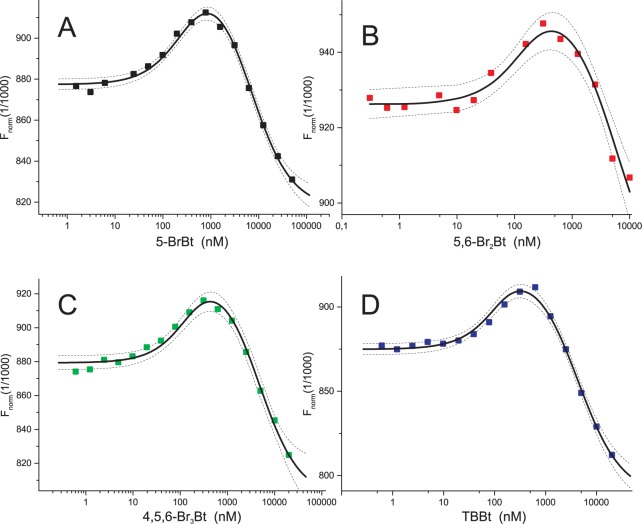
Representative MST pseudo-titration data for binding of halogenated benzotriazoles to hCK2α. Squares represent experimental data for 5-BrBt (A), 5,6-Br_2_Bt (B), 4,5,6-Br_3_Bt (C) and TBBt (D), solid lines follow the resulting model of two independent binding sites, and thin lines represents 95% confidence limits for this model. See also S2–S5 Figs for the complete set of experiments.

Dissociation constants were initially estimated with the aid of Microscale Thermophoresis. For each ligand a series of 6 pseudo-titration experiments were collected (S2–S5 Figs). As reported previously [13], some MST-derived data displayed the second inflection point at high ligand concentration, indicating the existence of a second weak binding site (Fig 2). Consequently, all data were reanalyzed according to the model of two independent binding sites [39]. The global optimization approach was used to estimate the common values for dissociation constants for the two binding sites (Table 1). Other experiment-specific parameters (i.e. asymptotic MST intensities for the *apo* protein, and its 1:1 and 1:2 complexes with a ligand) were fitted for each MST experiment independently (see Material and methods and S1 Fig).

**Table 1:** Aqueous solubility (C_w_) and thermodynamic parameters (K_d_, ΔG, ΔH, ΔS) for binding of halogenated benzotriazoles and the catalytic subunit of human protein kinase CK2 (hCK2α).

Parameter	Ligand			
	5-BrBt	5,6-Br_2_Bt	4,5,6-Br_3_Bt	TBBt
Aqueous solubility				
C_w_ (M) [36]	2.77·10^−3^	2.31·10^−4^	6.23·10^−5^	2.18·10^−4^
MST				
K_d1_ (nM)	246 ± 36	81 ± 22	83 ± 29	45 ± 11
ΔG_1_ (kJ/mol)	-37.7 ± 0.3	-40.5 ± 0.6	-40.4 ± 0.7	-41.9 ± 0.5
K_d2_ (μM)	6.3 ± 1.0	4.4 ± 0.5	4.6 ± 1.4	4.2 ± 0.4
ΔG_2_ (kJ/mol)	-29.7 ± 0.4	-30.6 ± 0.3	-30.5 ± 0.7	-30.7 ± 0.2
ITC (hCK2α titrated with the ligand)				
K_d_ (nM)	310 ± 89	1170 ± 270	990 ± 270	350 ± 100
ΔG (kJ/mol)	-37.1 ± 0.6	-33.9 ± 0.5	-34.3 ± 0.6	-36.8 ± 0.6
ΔH (kJ/mol)	-68 ± 3	-63 ± 2	-35 ± 2	-19 ± 1
ΔS (J/mol/K)	-102 ± 9	-97 ± 8	-2 ± 5	59 ± 4
Reverse ITC (ligand titrated with hCK2α)				
K_d_ (nM)	294 ± 73	46 ± 10	42 ± 17	39 ± 13
ΔG (kJ/mol)	-37.3 ± 0.5	-41.9 ± 0.5	-42.1 ± 0.8	-42.3 ± 0.7
ΔH (kJ/mol)	-70 ± 3	-71 ± 2	-40 ± 1	-26 ± 1
ΔS (J/mol/K)	-110 ± 10	-98 ± 6	9 ± 4	54 ± 3
Reverse ITC with MST-derived K_d_				
K_d1_ (nM)	246 ^x^	81 ^x^	83 ^x^	45 ^x^
ΔG_1_ (kJ/mol)	-37.7 ^x^	-40.5 ^x^	-40.4 ^x^	-41.9 ^x^
ΔH_1_ (kJ/mol)	-73 ± 5	-78 ± 13	-44 ± 2	-27 ± 5
ΔS_1_ (kJ/mol/K)	-118 ± 15	-126 ± 43	-12 ± 9	53 ± 18
K_d2_ (μM)	6.3 ^x^	4.4 ^x^	4.6 ^x^	4.2 ^x^
ΔG_2_ (kJ/mol)	-29.7 ^x^	-30.6 ^x^	-30.5 ^x^	-30.7 ^x^
ΔH_2_ (kJ/mol)	7 ± 23	11 ± 34	2 ± 9	6 ± 12
ΔS_2_ (kJ/mol/K)	123 ± 77	140 ± 114	109 ± 31	123 ± 41

Thermodynamic data were obtained using three alternative experimental methods (MST, ITC, reverse ITC), and additionally by the mixed approach, in which the model of two independent binding sites was applied using MST-derived binding affinities (K_d_) for ITC-based determination of heat of binding (ΔH).

^X^ these values were constrained based on the MST-derived binding affinities.

As expected, affinities determined for the strong binding site are close to the values estimated previously for a restricted range of ligand concentrations with the standard NanoTemper Software [13], in which only the model with one type of binding sites could be fitted. They are also consistent with inhibitory activities determined before for the same set of ligands [9]. The second, weak binding site may be attributed to the same phenomena that were observed for the closely related ligands, which bind at the interface between CK2α and CK2β subunits [40]. Since the affinity of this binding site is not high enough to inhibit the subunit interaction, the contribution of the additional weak binding site(s) may be regarded as physiologically negligible.

### Inconsistencies between the ITC and MST determined K_d_ values

**Fig 3:**
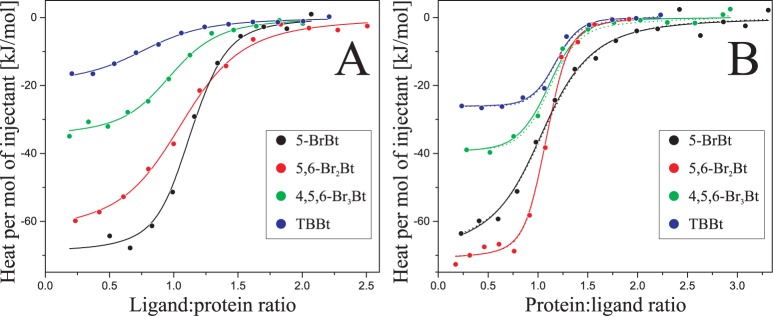
Isothermal Titration Calorimetry (ITC) data for binding of halogenated benzotriazoles to hCK2α. hCK2α is titrated with the ligand (A) and benzotriazoles are titrated with the protein (B). Circles represent integrated binding isotherms, whereas each solid line shows the fitted model of one type of binding sites. For comparison, the chopped lines in the panel B follow the model of two types of binding sites optimized with K_d_ values adopted from MST-derived binding affinities.

In parallel, Isothermal Titration Calorimetry experiments were carried out to determine the complete set of thermodynamic parameters describing the binding of all four ligands to hCK2α (Fig 3A). Although the ligand concentration in the syringe was always lower than the solubility limit [37], substantial discrepancies between ITC- and MST-derived data were identified (Table 1). The biding affinities determined with the help of the two methods were consistent only for 5-BrBt (246 nM for MST and 310 nM for ITC), solubility of which substantially exceeds the ligand concentration in the syringe. For all three other ligands, for which the syringe concentrations approached the solubility limits, the binding affinities determined with the aid of ITC were a few fold lower than those obtained with MST. Nevertheless, the observed differences could not be assigned directly to the ligand solubility. The presence of 2% DMSO substantially increases the solubility limits. The solubility of TBBt in pure DMSO is 65mM (http://www.sigmaaldrich.com/catalog/product/sigma/t0826), so it was expected and experimentally confirmed that for each of tested ligands at least 1 mM solution could be obtained in the buffer containing 2% of DMSO. The absence of the effective fast ligand disaggregation after injection to the calorimetric cell is also supported by the heat of ligand dilution, which remained constant for all subsequent injections (S6 Fig, gray lines). Any form of ligand oligomers/aggregates equilibrium should progressively affect both the heat of individual injections and the power baseline, which reflects the viscosity of the sample. However, none of these effects was observed.

The alternative explanation would be that, although in both methods the ligand concentrations did not exceed their apparent solubility, some of them may form soluble nano-aggregates. It should be noted that, although the same stock ligand solutions were used in both experiments, time elapsed between mixing of reagents and data acquisition was substantially different, which might have affected the apparent concentrations of the monomeric ligands accessible to the protein. The ligand and protein were mixed at least 10 min before each MST experiment. In contrast, the ligand was gradually injected in several steps into the protein containing cell during the ITC measurement. Consequently, the observed differences in affinities determined by both methods may be attributed to the difference in concentration of the monomeric form of the ligand that was available to the protein.

### Nano-aggregates detected by DLS and UV-VIS spectroscopy

**Fig 4:**
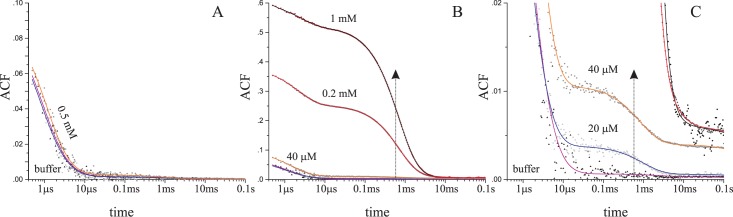
**DLS-derived autocorrelation function (ACF) measured for the decreasing concentration of 5-BrBt (A) and TBBt (B).** The shoulders located at 600 μs, denoted by the vertical arrow indicate 100 nm particles, detectable even for 20 μM TBBt solution (C).

To verify this hypothesis, the presence of virtually stable nano-aggregates was tested by the dynamic light scattering (DLS) measured for decreasing concentration of the ligand. DLS-derived autocorrelation function clearly proved that at 1 mM concentration TBBt forms nano-aggregates (Fig 4B) of a radius ~100 nm, while no such effect was observed for 5-BrBt (Fig 4A). The dilution of the TBBt sample resulted in a decrease of the population of the aggregates, however their average size remained unaffected, as clearly evidenced by a mid-point of the ACF function decay located at ~600 μs (Fig 4B). Interestingly, the aggregates could be identified even for the lowest 20 μM concentration of the TBBt solution (Fig 4C). Thus, the DLS data clearly confirmed that even at low concentration TBBt forms aggregates of the 100 nm radius. Unfortunately, the kinetics of disintegration of these nano-aggregates could not be directly followed by DLS, since at the lowest TBBt concentration the scattering by large particles was rarely identified, causing extreme fluctuations of the ACF. However, the raw SLS signal visibly decreased over time, indicating a slow (τ = 460 ± 40 s) decay of the aggregate population (S7A Fig).

**Fig 5:**
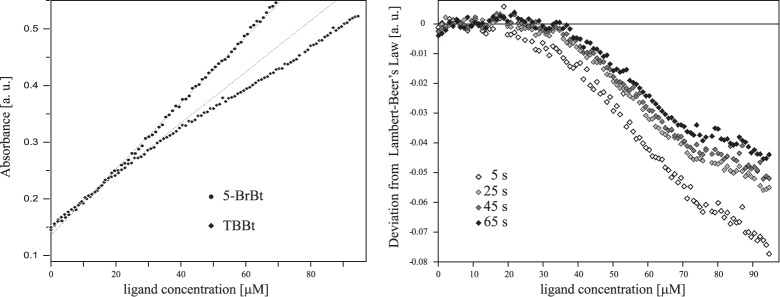
Deviation from the Lambert-Beer law observed upon stepwise increase of the solute concentration. The absorbance data are shown for 5-BrBt (A, circles) and TBBt (A, diamonds). The deviation from the low-concentration asymptote observed for TBBt slowly decreases over time (B).

Since the configuration of DLS apparatus precluded reliable analysis of the evolution of rare aggregates or small oligomers, their existence was further validated by the applicability of the Lambert-Beer law. Any deviations from the linear changes of the sample absorption upon increase of the solute concentration indicate oligomerization or even aggregation phenomena. Subtle deviations from the linear correlation between solute concentration and UV-absorbance have been observed (Fig 5A), with the distance to the low-concentration asymptote sensitive enough to support estimation of the upper limit of ligand concentration, at which monomeric form predominates. To exclude the non-linearity in Lambert-Beer law resulting from the inner filter effect of the spectrophotometer, the control experiment was done for 5-BrBt, for which the plot of concentration versus absorbance remained linear (Fig 5A).

A series of experiments performed for TBBt (Fig 5A) led to the estimation of the upper limit of ligand concentrations varying in the range of 20 μM to 30 μM. It must however be noted, that the ligands were used at higher concentration for the ITC injections (see Material and methods). Thus, initially the protein might have been exposed to ligand nano-aggregates, which after injection to the sample cell slowly spontaneously disappeared. The spectroscopic data clearly indicates that TBBt oligomerizes or aggregates at concentrations exceeding 20–30 μM. (Fig 5). Since 5,6-dibromobenzotriazole and 4,5,6-tribromobenzotriazole display similar aqueous solubility [37], it may be generalized that analogous effect influences the ITC measurements carried out for the two ligands, as well.

### Time dependent decay of the aggregates and its implications for the protein-ligand assays

Interestingly, the deviation from the Lambert-Beer law was found to be time-dependent (Fig 5B). The difference between the measured and expected absorbance systematically decreases 25, 45 and 65 s after solute dilution, and the concentration range at which monomeric form predominates increases. This observation demonstrates that soluble aggregates injected to the buffer disappear with a time-scale of several hundreds of seconds. This effect was additionally monitored for 40 μM solution of TBBt. The observed increase of TBBt absorption at 291 nm (S7B Fig) is indicative of the increasing population of the solute monomeric form (τ = 670 ± 40 s), while a slight decrease of absorption at 483 nm reflects the decay of soluble aggregates that scatter the light (S7C Fig). Thus, the kinetics of the decay of non-monomeric ligand forms determines the actual concentration of its monomeric form, affecting the formation of protein-ligand complexes.

In MST experiment, the samples were routinely prepared 10–20 minutes before the actual measurement, so the ligand was mostly in the monomeric form, at least at low concentrations applicable for determination of the binding affinity. However during ITC measurement, the series of small concentrated aliquots of ligand were injected directly into the protein solution, and only the heat power released upon rapid mixing of reagents after injection can be effectively measured. The other heat effects accompanying slowly appearing monomers (τ = 400–700 s) and their interaction with the protein, are hidden in the thermal noise (noise level of 2.5 nW is declared in the supplier specification). Therefore, the protein-ligand affinities determined with the aid of ITC may be systematically underestimated. The same effect may bias the biochemical studies of inhibitory activity, in which a concentrated inhibitor solution is added to the protein sample immediately before the activity test. Such hypothesis is supported by the finding that the “pre-incubation” of TBBt with CK2α substantially improves its inhibitory activity in the enzymatic assay [38].

### Ligand aggregation avoided by the use of reverse ITC

**Fig 6:**
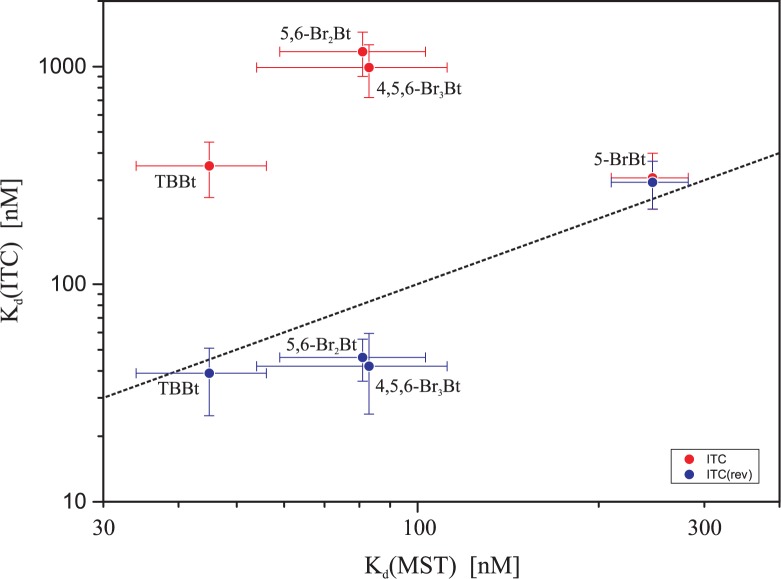
Correlation between MST- and ITC-derived binding affinities determined for complexes of halogenated benzotriazoles with hCK2α. K_d_(ITC), were obtained with ITC experiment, in which either inhibitor (red) or protein (blue) was used as a titrant. Vertical and horizontal bars represent standard deviation (MST) and 67% confidence intervals (ITC), respectively.

It should be stressed that the reported problems may be avoided, for well-soluble non-aggregating preferably monomeric proteins, by the application of reverse ITC experiment, in which the protein is used as a titrant (Fig 3B). According to our data, the values of dissociation constants obtained in such experiment agree with the MST-derived ones (Fig 6).

Binding of all ligands was exothermic, but two of them (TBBt and 4,5,6-Br_3_Bt) displayed favorable entropic contribution. It may be speculated that, according to the review of Battistuta *et al*. [41], 4,5,6-Br_3_Bt and TBBt bind to hCK2α in the orientation similar to the one of TBBt in the complex with maize CK2α (pdb1j91 [7]), while two other ligands more likely occupy the location proximal to that of TBBz in the complex with the kinase (pdb2oxy [41]). The binding to the weak site was entropically driven for all studied ligands.

It should be noted that the estimated heat of binding and entropic contribution, should, at least for TBBt and 4,5,6-Br_3_Bt (for which the anionic forms predominate), take into account the heat of buffer ionization, which is relatively high for Tris-HCl (47.5 kJ/mol). Unfortunately, the small heat of interaction between TBBt and 4,5,6-Br_3_Bt precluded the application of commonly used buffers with moderate ionization enthalpy (e.g. Hepes: 20.4 kJ/mol; Tricine: 31.4 kJ/mol or TES: 32.1 kJ/mol [42]).

**Fig 7:**
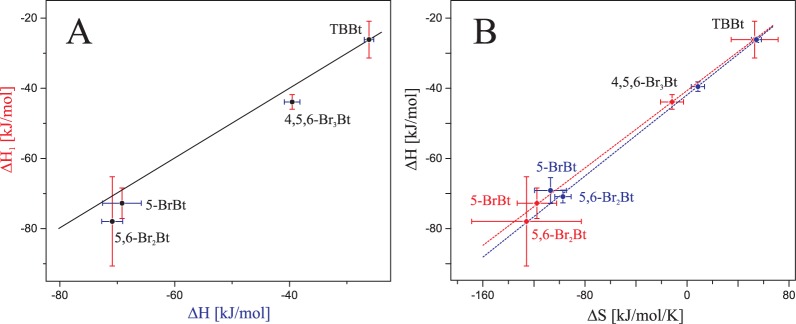
Correlation between thermodynamic parameters determined from the reverse ITC experiment. The data were estimated using the model of either one (blue) or two (red) types of binding sites, in which both K_d_ values were adopted from MST data. Panel A shows the correlation of the heat of binding to the strongly binding site determined by both methods, whereas in panel B the entropy vs. enthalpy relation for the strongly binding site is shown. Vertical and horizontal bars represent 67% confidence intervals.

It should also be noted that, due to limited reagents solubility, weekly binding sites identified by MST could not be effectively sampled by calorimetric methods, even using reverse ITC. However, we succeeded in fitting both heats for dissociation, by keeping the K_d_ values constrained with the MST-derived affinities. The resulting model agrees with the experimental data (Fig 3B, chopped lines), but the analysis of the residual variance indicated that it cannot be regarded as a better fit than the model with one type of binding sites only. All ITC-derived thermodynamic parameters are summarized in Table 1. Interestingly, apart from the small differences in binding affinities, the heat of binding to the strong binding site remains identical for both types of analysis of the reverse ITC data (Fig 7A). Moreover, entropy vs. enthalpy relation is almost identical in both cases (Fig 7B), clearly indicating that the reverse ITC experiment, in which the protein is used as a titrant, extends the applicability of the Isothermal Titration Calorimetry for hydrophobic ligands.

## Conclusions

According to our analysis, ITC-derived dissociation constants may be substantially overestimated due to the limited titrant solubility resulting in the formation of nano-aggregates. The same concerns apply to biochemical assays, in which the concentrated ligand sample is added immediately before the enzymatic assay. Fortunately, the inaccuracy in the K_d_ determination may in many cases be avoided by the use of reverse ITC experiment, in which a protein is applied as a titrant. Alternatively, the ITC-derived thermodynamic data should routinely be verified by an alternative method such as Microscale Thermophoresis.

## Supporting information

S1 Fig Idealized MST pseudo-titration data (diamonds) interpreted according to the model of two independent binding sites (black curve). Thin lines demonstrate relations expected for the effects associated with binding of the first (red) and second (blue) ligand molecule, while dotted horizontal lines denote levels of the MST signals estimated for *apo*, 1:1 and 1:2 protein forms, respectively.
(PDF)

S2 Fig MST-derived pseudo-titration data for binding of TBBt by hCK2α. Circles represent experimental data, solid line follows the model of two independent sites, and thin lines represent 95% confidence limits for the model. Two dissociation constants (45±11 nM and 4.2±0.4 μM) were fitted globally, while the signals characterizing three protein states (*apo*, 1:1 and 1:2 complexes) were for each experiment estimated independently.
(PDF)

S3 Fig MST-derived pseudo-titration data for binding of 4,5,6-Br_3_Bt by hCK2α. Circles represent experimental data, solid line follows the model of two independent sites, and thin lines represent 95% confidence limits for the model. Two dissociation constants (83±29 nM and 4.6±1.3 μM) were fitted globally, while the signals characterizing three protein states (*apo*, 1:1 and 1:2 complexes) were for each experiment estimated independently.
(PDF)

S4 Fig MST-derived pseudo-titration data for binding of 5,6-Br_2_Bt by hCK2α. Circles represent experimental data, solid line follows the model of two independent sites, and thin lines represent 95% confidence limits for the model. Two dissociation constants (81±22 nM and 4.4±0.4 μM) were fitted globally, while the signals characterizing three protein states (*apo*, 1:1 and 1:2 complexes) were for each experiment estimated independently.
(PDF)

S5 Fig MST-derived pseudo-titration data for binding of 5-BrBt by hCK2α. Circles represent experimental data, solid line follows the model of two independent sites, and thin lines represent 95% confidence limits for the model. Two dissociation constants (246±36 nM and 6.3±1.0 μM) were fitted globally, while the signals characterizing three protein states (*apo*, 1:1 and 1:2 complexes) were for each experiment estimated independently.
(PDF)

S6 Fig Representative heat power plots obtained with for ITC. Titrations of either buffer (gray lines) or hCK2α (color lines) with 5-BrBt (A), 5,6-Br_2_Bt (B), 4,5,6-Br_3_Bt (C) and TBBt (D).
(PDF)

S7 Fig Time dependence of TBBt disaggregation. The process occurring after rapid dilution of 500 μM sample was followed by SLS (A), TBBt absorbance at 291 nm (B), and apparent solvent absorbance at 483 nm (C).
(PDF)
