# Supplementary material for: ITC-derived binding affinity may be biased due to titrant (nano)-aggregation. Binding of halogenated benzotriazoles to the catalytic domain of human protein kinase CK2
Source: PLoS One. 2017 Mar 8;12(3):e0173260. doi: 10.1371/journal.pone.0173260 (PMC5342230; doi:10.1371/journal.pone.0173260)
Supplement: S1 Fig — Thin lines demonstrate relations expected for the effects associated with binding of the first (red) and second (blue) ligand molecule, while dotted horizontal lines denote levels of the MST signals estimated for apo, 1:1 and 1:2 protein forms, respectively. (PDF) [file pone.0173260.s001.pdf]

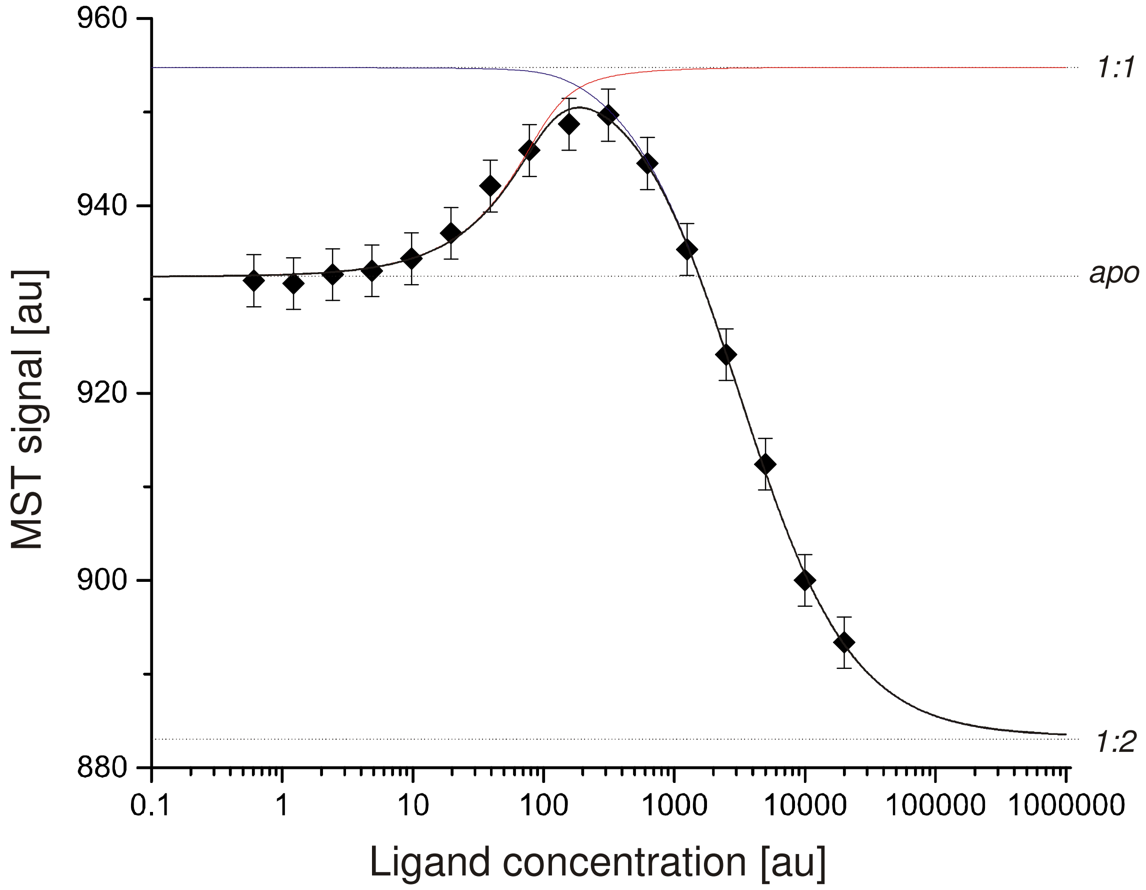

S1 Fig. Idealized MST pseudo-titration data (diamonds) interpreted according to the model of two independent binding sites (black curve). Thin lines demonstrate relations expected for the effects associated with binding of the first (red) and second (blue) ligand molecule, while dotted horizontal lines denote levels of the MST signals estimated for apo, 1:1 and 1:2 protein forms, respectively.
