# Supplementary material for: ITC-derived binding affinity may be biased due to titrant (nano)-aggregation. Binding of halogenated benzotriazoles to the catalytic domain of human protein kinase CK2
Source: PLoS One. 2017 Mar 8;12(3):e0173260. doi: 10.1371/journal.pone.0173260 (PMC5342230; doi:10.1371/journal.pone.0173260)
Supplement: S4 Fig — Circles represent experimental data, solid line follows the model of two independent sites, and thin lines represent 95% confidence limits for the model. Two dissociation constants (81±22 nM and 4.4±0.4 μM) were fitted globally, while the signals characterizing three protein states (apo, 1:1 and 1:2 complexes) were for each experiment estimated independently. (PDF) [file pone.0173260.s004.pdf]

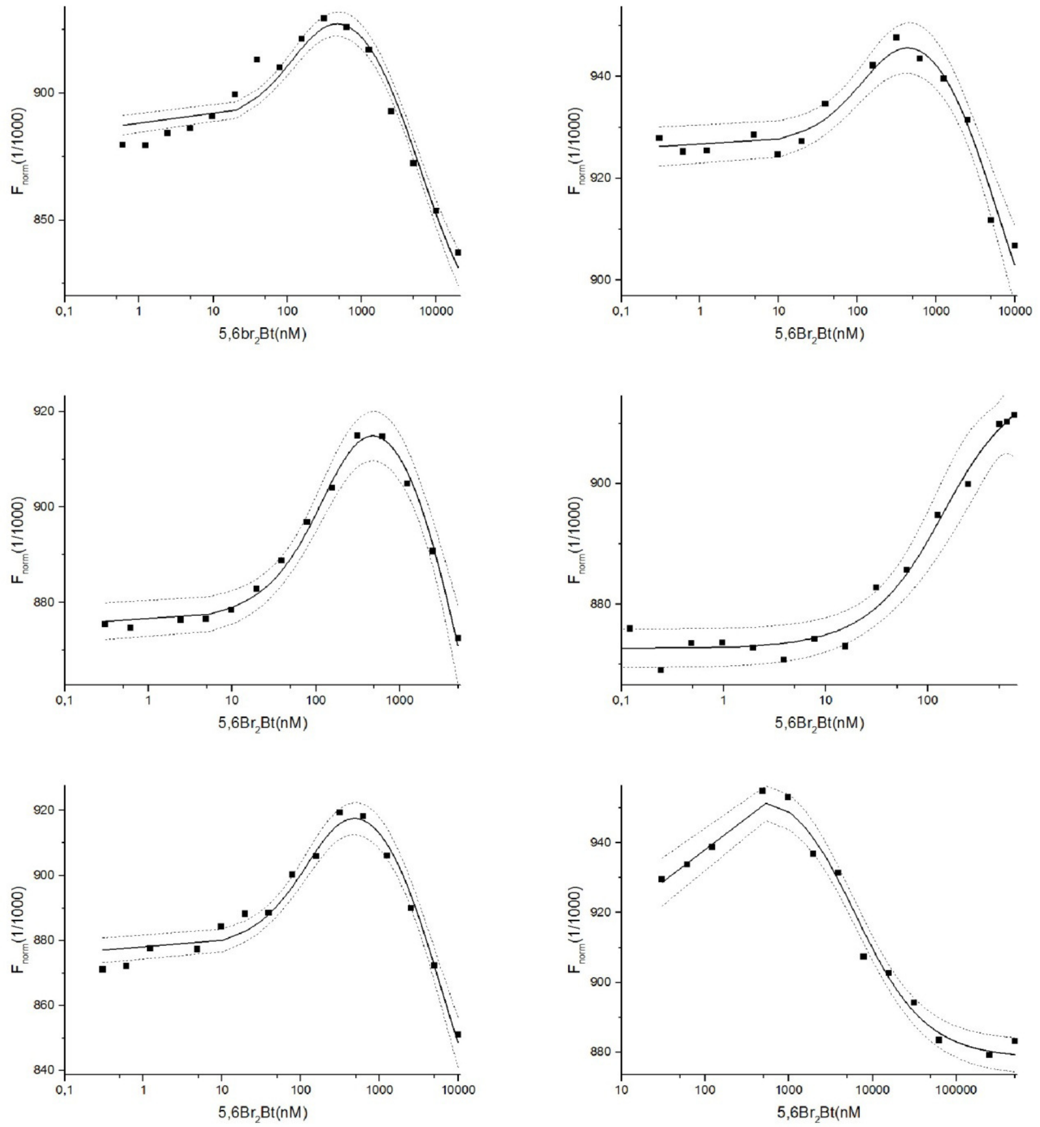

S4 Fig. MST-derived pseudo-titration data for binding of 5,6Br$_2$Bt by hCK2α. Circles represent experimental data, solid line follows the model of two independent sites, and thin lines represent 95% confidence limits for the model. Two dissociation constants (81±22 nM and 4.4±0.4 μM) were fitted globally, while the signals characterizing three protein states (*apo*, 1:1 and 1:2 complexes) were for each experiment estimated independently.
