# Supplementary material for: ITC-derived binding affinity may be biased due to titrant (nano)-aggregation. Binding of halogenated benzotriazoles to the catalytic domain of human protein kinase CK2
Source: PLoS One. 2017 Mar 8;12(3):e0173260. doi: 10.1371/journal.pone.0173260 (PMC5342230; doi:10.1371/journal.pone.0173260)
Supplement: S6 Fig — Titrations of either buffer (gray lines) or hCK2α (color lines) with 5-BrBt (A), 5,6-Br2Bt (B), 4,5,6-Br3Bt (C) and TBBt (D). (PDF) [file pone.0173260.s006.pdf]

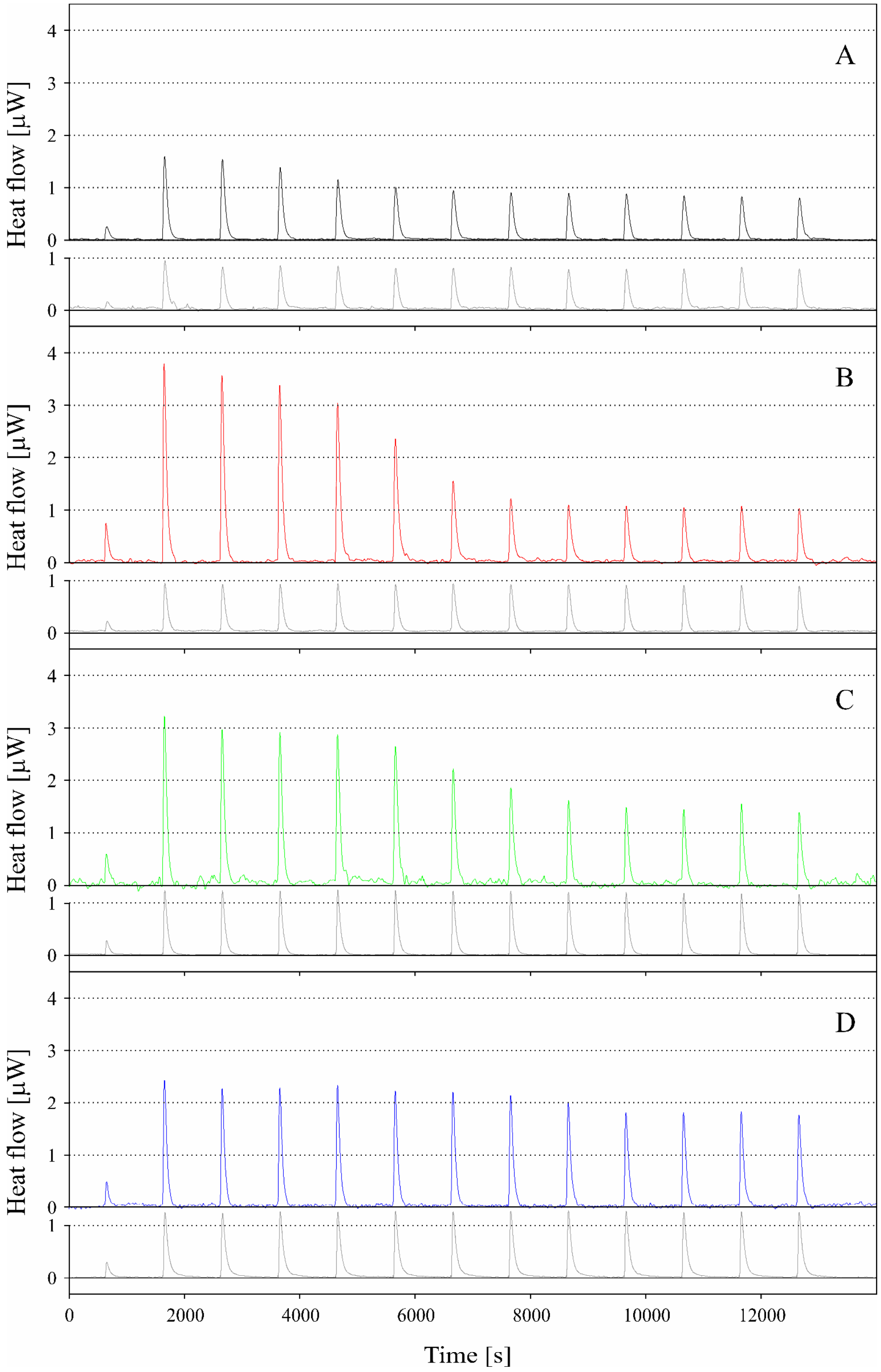

S6 Fig. Representative heat power plots obtained with ITC. Titrations of either buffer (gray lines) or hCK2α (color lines) with 5-Br2Bt (A), 5,6-Br2Bt (B), 45,6-Br3Bt (C) and TBBt (D).
