# Supplementary material for: ITC-derived binding affinity may be biased due to titrant (nano)-aggregation. Binding of halogenated benzotriazoles to the catalytic domain of human protein kinase CK2
Source: PLoS One. 2017 Mar 8;12(3):e0173260. doi: 10.1371/journal.pone.0173260 (PMC5342230; doi:10.1371/journal.pone.0173260)
Supplement: S7 Fig — The process occurring after rapid dilution of 500 μM sample was followed by SLS (A), TBBt absorbance at 291 nm (B), and apparent solvent absorbance at 483 nm (C). (PDF) [file pone.0173260.s007.pdf]

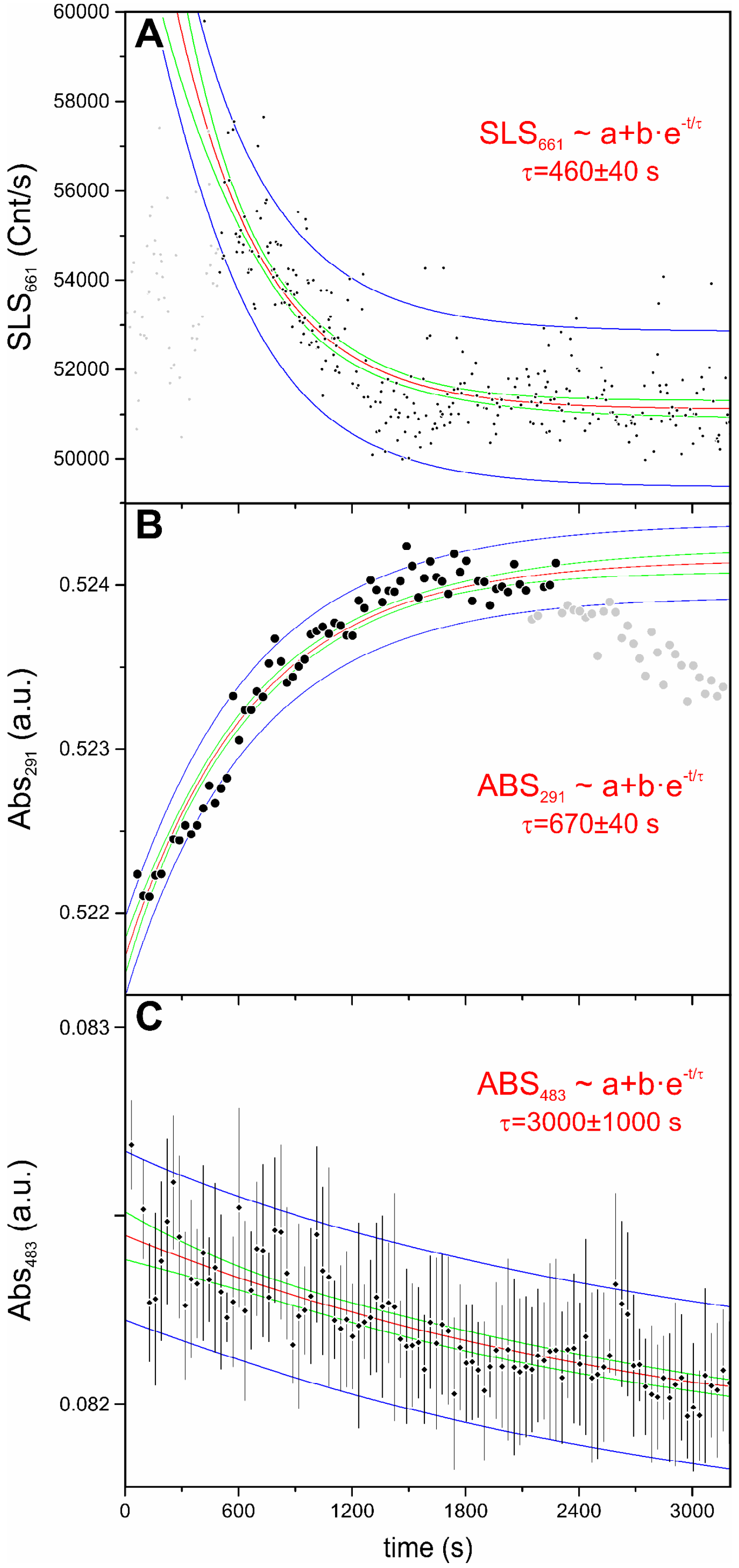

S7 Fig. Time dependence of TBBt disaggregation. The process occurring after rapid dilution of 500 μM sample was followed by SLS (A), TBBt absorbance at 291 nm (B), and apparent solvent absorbance at 483 nm (C).
